# An improved bone transport surgical method for treating chronic ischemic ulcers (thromboangiitis obliterans)

**DOI:** 10.3389/fsurg.2022.859201

**Published:** 2022-08-19

**Authors:** Liang Zhao, Yu Lei, Mengru Pang, Zairong Wei

**Affiliations:** ^1^ Department of Burns and Plastic Surgery, The Affiliated Hospital of Guizhou Medical University, Guiyang, China; ^2^ Department of Burns and Plastic Surgery, The Affiliated Hospital of Zunyi Medical College, Zunyi, China

**Keywords:** thromboangiitis obliterans, ischemic ulcers, chronic wound, bone transport technique, improved surgical method

## Abstract

**Introduction:**

The chronic ischemic injury of the upper/lower limbs caused by thromboangiitis obliterans (TAO, Buerger's disease) is difficult to heal, leading to high morbidity and amputation risk, seriously lowering the quality of life of patients. So far, the pathogenesis of this disease is still not clear, and there are still no effective therapeutic approaches. Here, we first use an improved bone transport technique to treat TAO-related foot ulcers and achieve good therapeutic effects.

**Materials and Methods:**

In this report, 22 patients met the inclusion criteria, and we provide an improved bone transport technique to repair TAO-related chronic lower limb wounds, which have a minimally surgical incision and a satisfying surgical field.

**Results:**

The improved bone transport technique resulted in TAO-related chronic lower extremity wound healing in most patients (18, M:F 16:2) within the first treatment cycle. All wounds healed completely after two treatment cycles. After these cycles, the cold sensation in the patients’ feet was significantly relieved, and the rest pain in the lower extremities was significantly relieved (Visual Analog Scale, *P* < 0.0001). Furthermore, the Laser Doppler flowmeter showed that the blood perfusion and percutaneous oxygen pressure of the affected foot were higher than in preoperation (*P* < 0.0001). To conclude, bone transport technology is available for the refractory wounds of the extremity, which may promote healing by increasing blood circulation and tissue oxygen supply.

**Conclusions:**

In summary, the improved surgical method of the bone transport technique is worth considering in the treatment of thromboangiitis obliterans–related foot ulcers.

## Background

Thromboangiitis obliterans (TAO) is classified as a vasculitis because of its acute inflammation involving all layers of the vessel wall. This inflammatory vascular disease mainly affects small and medium-sized arteries and veins. Tobacco use is related to disease occurrence and re-occurrence (1, 2). This disease is more common in men, and women account for approximately only 22.8% according to statistics (3). Distal ischemic syndrome leads to chronic ischemic ulcers of the limbs, and high amputation rates are the most important feature of this disease.

TAO was first reported in 1879 in Vienna. It occurs all over the world and has a high prevalence in the Mediterranean, the Middle East, and India (4). When TAO-related ischemic lesions occur, there is no preferred treatment option, and it is reported that the related amputation rate is up to 75% within 3–10 years of follow-up of TAO patients (the main amputation rate is 31%) (5). Minor amputations contribute to the main proportion. Also, the high amputation rates of young patients have led to a huge economic and social welfare burden on families. Therefore, finding an effective treatment for this disease is necessary (1).

Tibial cortex transverse transport (TTT) has received increased attention due to its application in the treatment of the diabetic foot. The application of this technology has helped many diabetic foot patients avoid amputation (6). It has been reported that the ulcer healing rate and limb salvage rate of the surgical group are significantly higher than those of the non-surgical group within 6 months. The simple operation, low cost, and good curative effect are its advantages (7, 8).

TAO patients are usually young low-status men, and the health of the limbs is not only related to mental pressure but also closely related to the future economic income of the entire family. In order to help patients avoid amputation, we studied and improved transport techniques over the last 2 years.

**Figure 1:**
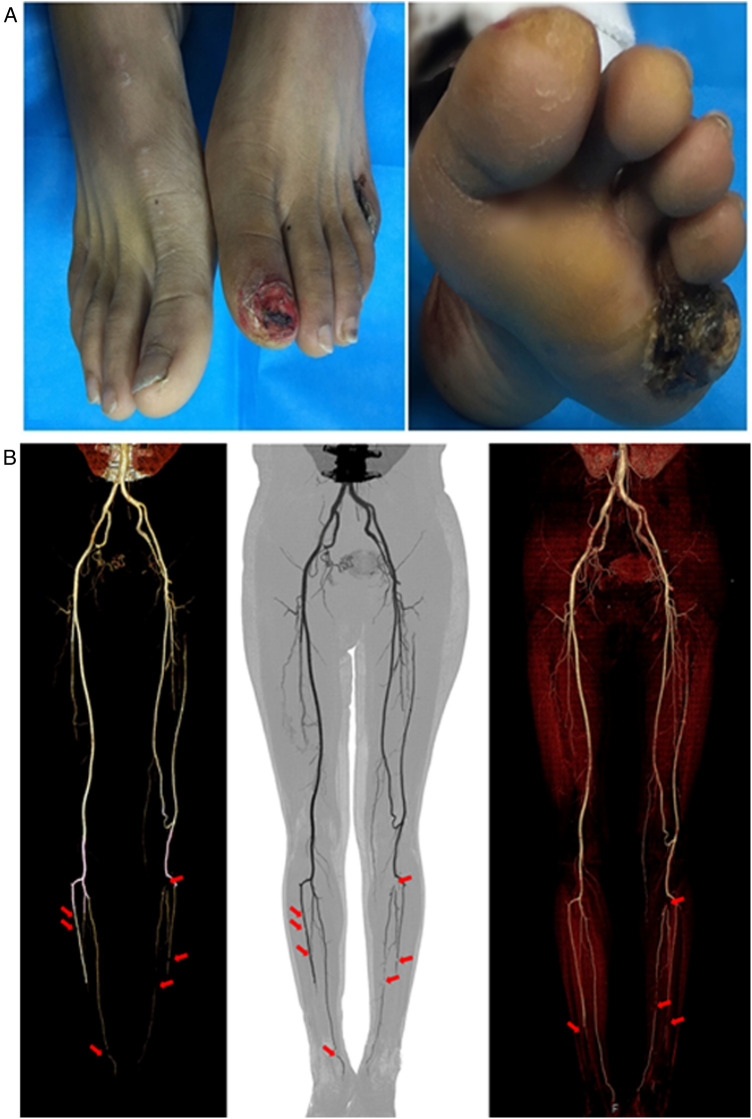
(**A**) A photograph of the patient before treatment, showing his lower limb chronic ischemic ulcer wound. (**B**) Computed tomographic arteriography (CTA) shows vasculitis of large and medium arteries noninvasively (red arrows).

So far, our team has performed this surgical treatment for 22 TAO patients in total, and 20 of them have obtained a good therapeutic effect (Figure 1A). This article will show how we use an improved surgical method of the bone transport technique to treat TAO-related lower limb chronic ischemic ulcer wounds.

## Materials and methods

### Ethics statement

The study protocol was approved by the Ethics Review of the Affiliated Hospital of Zunyi Medical College, 2016, No.7 and the Ethics Committee of the Affiliated Hospital of Guizhou Medical University (approval no. [2020] 281). Consent for publication was obtained from all patients and their families.

### Patients

Patients suffering from TAO who meet the following inclusion criteria were assessed (Tables 1, 2; Figure 1): (1) have been diagnosed with TAO for more than 2 years; (2) symptoms of lower extremity soreness, accompanied by lower extremity ulcers over 4 weeks; (3) continuous tobacco use over 2 years; (4) positive angiographic imaging; and (5) exclude diabetes, trauma, and other diseases that may cause chronic ulcers in the lower limbs. Exclusion criteria included: (1) traumatic vascular injury; (2) hypertension; (3) coronary heart disease; (4) hyperlipidemia; and (5) diabetes. A total of 22 patients with 26 affected limbs were included, and two of the affected limbs were ultimately amputated.

**Table 1:** Inclusion criteria for improved tibial cortex transverse transport.

Inclusion criteria
(1) Have been diagnosed with TAO for more than 2 years.
(2) Symptoms of lower-extremity soreness, accompanied by lower-extremity ulcers over 4 weeks
(3) Continuous tobacco use over 2 years
(4) Positive angiographic imaging
(5) Exclude diabetes, trauma, and other diseases that may cause chronic ulcers in the lower limbs

**Table 2:** Basic information of the patient.

Patient information
Sex (male/female)	20/2
Age (year)	40 ± 8
Smoking history (year)	22 ± 8
Average number of cigarettes (per day)	23 ± 7
Affected limb (left/right/both)	5/7/10
Follow-up (month)	14.2 ± 6.3

### The protocols of the improved bone transport technique

**Figure 2:**
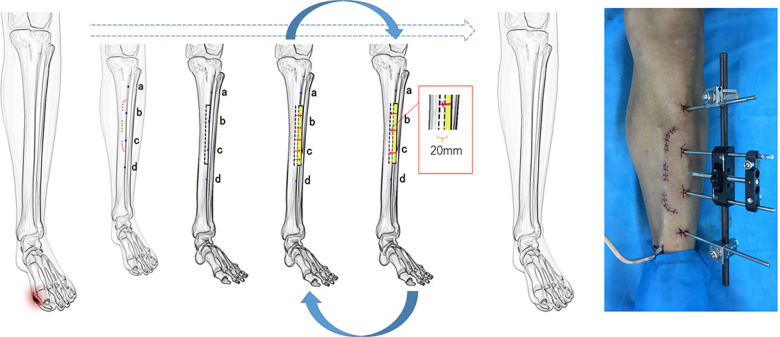
Schematic diagram of the surgical operation process, showing the surgical operation design and the installation of external fixation systems. The red dotted line indicates the operative incision. The black dotted line indicates the range of the bone flap. The blue dots (a, b, c, and d) indicate the position of the four metal pins. One treatment cycle is approximately 20 days.

All patients had a stable internal environment, and the anesthesiologist determined that surgery could be performed after evaluation. Subarachnoid anesthesia and spinal anesthesia or epidural anesthesia were used. The patients were placed in a supine position. After the anesthesia took effect, metal bone needles were first designed to be placed at a, b, c, and d of the lower limbs (the upper 2/3 of the tibia, equidistant), and the skin and fascia were cut with a sharp knife, respectively. Vascular forceps were used to separate the subcutaneous tissue, the periosteum to the cortical bone. A protective drill bit sleeve was inserted perpendicular to the tibial crest, and holes were drilled under the assistance of normal saline to cool down, and then a metal bone needle was installed. The skin, subcutaneous tissue, deep tissue, and the periosteum were incised along the crescent-shaped incision, and the periosteum was peeled off with a periosteal peeler to expose the tibia (approximately located at the medial 0.5 cm between the proximal 1/3-distal 1/3 of the tibia, and the distance between each incision is greater than 2 cm). Under normal saline–assisted cooling, the electric drill was used to drill holes along the inner half edge of the designed bone flap, and the osteotome cut the cortical bone (approximately 2 × 10 cm in size) to separate the bone flap from the tibial trunk. The periosteum at the lateral edge of the bone flap was not cut. After anatomical reduction of the bone flap, a single-arm integrated orthopedic external fixation bracket system (Tianjin Xinzhong Medical Instrument Co., Ltd.) was installed, and the external fixation bracket system was adjusted to be stable under the irradiation of a C-arm x-ray machine. The wound surface was washed with normal saline, and the periosteum was sutured after confirming that there was no active bleeding. A negative pressure drainage tube was placed on the upper edge of the periosteum. After fixation, a vacuum negative pressure gauge (0.02–0.04 MPa) was connected (Figure 2). Tissue and skin were bandaged with sterile dressings. There was no need to use tourniquets and electrocautery during the operation.

### Statistical analysis

Statistical analysis was performed using one-way analysis of variance to compare the experimental groups with the control group and using the paired *t*-test to test for differences between the paired groups. All statistical analyses were performed using SPSS software (IBM Corp., Armonk, NY, USA). A value of *P* < 0.05 was considered statistically significant.

## Results

The condition of 20 patients improved significantly after the operation, and 2 patients with unilaterally affected limbs progressed postoperatively and underwent amputation finally; no surgical site infection occurred in all patients after the operation; 24 of the 26 affected limbs were successfully treated, and the limb salvage rate was 92.3%.

### Rest pain

**Figure 3:**
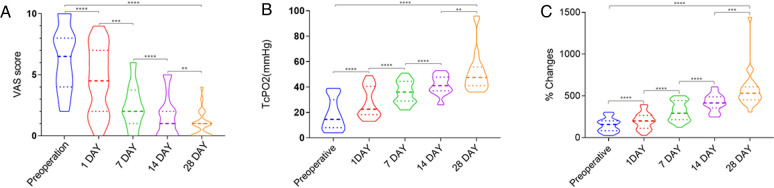
(**A**) Visual Analog Scale (VAS) pain score. The patients felt that the pain was significantly relieved after the operation (1 day, 7 days, 14 days, 28 days) compared with the preoperative period. (**B**) Transcutaneous oxygen pressure (TcPO 2). (**C**) % changes in blood perfusion. After the operation, the transcutaneous oxygen pressure and blood perfusion of the affected limb significantly increased. ****, *P* < 0.0001; ***, *P* < 0.001; **, *P* < 0.01; *, *P* < 0.05.

The rest pain of the affected limb was significantly relieved in 20 patients who showed improvement after the operation (Figure 3A). The VAS scores before operation and at 1, 7, 14, and 28 days after operation were (6.3 ± 2.6), (4.6 ± 2.7), (2.5 ± 1.7), (1.6 ± 1.7), and (0.9 ± 1.1), respectively, and these scores were significantly lower than those before operation (*P* < 0.05).

### Degree of ischemia

The ischemia in the affected limb significantly reduced in 20 patients. The transcutaneous oxygen partial pressures before and 1, 7, 14, and 28 days after surgery were (18.3 ± 12.3), (27.8 ± 12.1), (35.7 ± 8.7), (41.5 ± 7.3), and (51.4 ± 15.7) mmHg (1 mmHg = 0.133 kPa), respectively. They were significantly higher at 1, 7, 14, and 28 days after the operation than before the operation (*P* < 0.0001) (Figure 3B). The percentages of temperature-controlled blood perfusion before and 1, 7, 14, and 28 days after surgery were (152.4 ± 82.1)%, (202.0 ± 100.3)%, (316.4 ± 116.3)%, (421.1 ± 99.5)%, and (573.8 ± 221.4)%, and in the postoperative 1, 7, 14, and 28 days, they were significantly higher than those before operation (*P* < 0.0001) (Figure 3C).

### Wound healing

**Figure 4:**
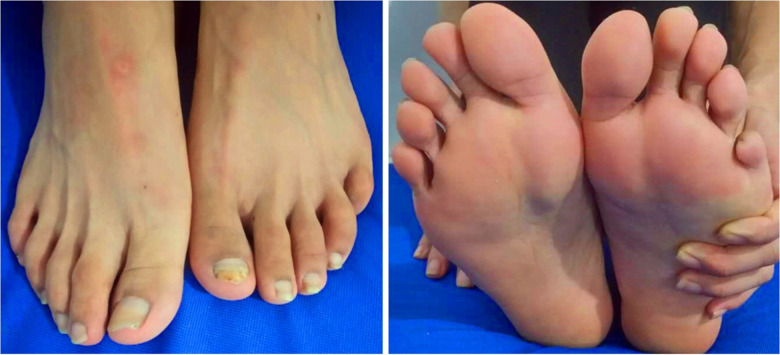
A photograph of the patient after treatment, showing his healed lower limb.

The 20 improved patients had various preoperative wound sizes and different degrees of ischemic ulcers of the affected limbs before surgery. The basic process of ischemic ulcer wound healing is similar. Routine dressing changes and debridement were accepted by some patients after surgery. The improved bone transport technique resulted in TAO-related chronic lower extremity wound healing in most patients (18, M:F 16:2) within the first treatment cycle. In total, 24 wounds healed completely after two treatment cycles (Figure 4). During the follow-up (14.2 ± 6.3) months after the operation, the ischemic ulcer or gangrenous wounds of the affected limbs healed, no new wounds formed, the skin temperature breached the normal level, and the sensory and motor functions of the affected limbs became normal.

### Amputee

One patient with a unilateral affected limb had a mild relief of rest pain on the first day after the operation, a slightly increased percutaneous oxygen partial pressure and temperature-controlled blood perfusion percentage, and a slightly improved skin temperature and color compared with the preoperative period. A total of 1 week after the operation, the gangrene of the affected limb gradually aggravated, and the pain at rest aggravated. The patient opted for amputation. One patient with a unilateral affected limb had been treated with drugs, interventional surgery, and bone marrow stem cell transplantation before surgery. The rest pain of the affected limb did not relieve significantly, the gangrenous wound did not shrink, and the wound surface was repeatedly infected. After the operation, the rest pain did not relieve. The area of gangrene did not expand significantly, and although the exudation in the necrotic area decreased, the healing process was slow, and the patient chose amputation for treatment.

## Discussion

The bone transverse transport technique has achieved good clinical results in the treatment of difficult-to-heal limb ulcers, especially in the treatment of the diabetic foot. Microcirculation disorders play an important role in the formation of ulcers. It was reported that the law of tension stress could activate and enhance the regenerative potential of living tissues, leading to the growth or regeneration of muscles, fascia, blood vessels, and nerves simultaneously (9). The distraction osteogenesis process is usually accompanied by rich vascular network formation. This may be the core mechanism for achieving the best results from the bone transverse transport technique in the treatment of diabetic foot ulcers. Diabetic foot ulcers and TAO-related foot ulcers have a certain similarity with regard to the formation mechanism: both have lower limb blood supply disorder. In theory, applying this technology to TAO-related lower extremity ulcers can also help achieve good results. Due to the general poor blood supply in these patients, and to obtain a more stable treatment effect, we have slightly improved the original surgical technique.

Compared with the traditional technology, three surgical improvements have been effected to the bone transport technique to obtain the greatest therapeutic benefits. Firstly, the shape of the operative incision is designed like a crescent consisting of three discrete parts. Compared with a long and continuous operative incision, the three-part crescent operative incision not only ensures a clear surgical field of vision, but also has a small surgical incision, less tissue damage, and faster wound healing. Secondly, compared with the traditional way in which the bone flap is located on the inside of the tibia away from the tibial crest (the lateral end 2 cm next to the tibial crest), we chose the bone flap in the tibial crest (Figure 2). The location change makes the operation area relatively superficial, so the surgical field is exposed clearly without obvious skin stretch and the bone flap is easier to obtain, the effect on muscle function is relatively small, and the wound cannot crack easily, which means that the wound can heal easily. During surgery, we only open the periosteum of the medial margin of the bone flap when making the bone flap to separate it from the tibial trunk. By using the bone knife, we can succeed in obtaining the bone flap and retain the integrity of the periosteum of the lateral margin of the bone flap. With this, there is maximum preservation of the vascular supply of the bone flap. Perfusion of the tibia is done by using three systems of vessels, one of them is the nonpenetrating periosteal vessel typical of the diaphysis (10). Reducing the damage of the periosteum can decrease the rate of osteonecrosis of the bone flap. More importantly, through this adjustment, the external fixation frame originally placed on the inner side of the tibial trunk can be placed on the front side of the tibial trunk, which greatly improves the comfort and portability of the fixation frame after placement, which is beneficial for the patient to perform lower-extremity activities. It is more conducive to lower limb circulation and wound healing. Thirdly, we placed a negative pressure drainage tube on the upper border of the periosteum. The vacuum sealing drainage with normal saline continuous irrigation is the most important improvement (The negative pressure is approximately 0.02–0.04 MPa). Its importance is reflected in two aspects. On the one hand, congestion and exudate can be eliminated in time (11). This makes it possible to avoid the occurrence of subperiosteal and subcutaneous hematomas and reduce the complications of tissue necrosis, surgical incision infection, and osteomyelitis. On the other hand, the topical negative pressure can produce a positive stimulating effect on vessel proliferation and improve the topical microcirculation (12). All of these help improve limb ischemic symptoms.

The law of tension stress indicates that the distraction area has three times greater blood vessel volume ratio than the intact contralateral side, and the peak blood flow is up to 10 times that of the normal side (11, 12). On the distracted side, dense new blood vessels formed along the distraction direction, and the ascending and descending arteries of the bone marrow also significantly expanded (12). This means not only will the blood supply of the surgical area increase, but the blood perfusion of the entire surgical side will also significantly increase. It is reported that this change in blood flow can last up to 17 weeks. This gives sufficient time for the healing of chronic ischemia-related wounds, such as diabetic foot and TAO-related limb ulcers. Also, the mesenchymal stem cells (MSCs) and mesenchymal stem cell–derived exosomes were found to have enhanced angiogenesis and bone healing by increasing the expression of vascular endothelial growth factor (VEGF) and also enhanced human umbilical vein endothelial cell proliferation, migration, and tube formation (13–15). The role of circulating MSCs in bone healing may play an important role in re-establishing the vasculature for oxygen delivery. Moreover, theoretically, the secreted cytokines and growth factors, such as IL-1 to IL-6, platelet-derived growth factor, VEGF, and bone morphogenetic protein from platelets, inflammatory cells, and macrophages caused by the fracture may all act on the limb wounds through blood circulation. This means that this technique is likely to be effective for most difficult-to-heal wounds of the limbs. This technology may provide a treatment plan for most chronic wounds.

## Conclusions

In summary, the improved surgical method of the bone transport technique is worth considering in the treatment of TAO-related lower limb chronic ischemic ulcer wounds.

## Data Availability

The original contributions presented in the study are included in the article/Supplementary Material, further inquiries can be directed to the corresponding author/s.
